# Cardiovascular Risk in Myositis Patients Compared to the General Population: Preliminary Data From a Single-Center Cross-Sectional Study

**DOI:** 10.3389/fmed.2022.861419

**Published:** 2022-05-03

**Authors:** Sabina Oreska, Hana Storkanova, Jaroslav Kudlicka, Vladimir Tuka, Ondrej Mikes, Zdislava Krupickova, Martin Satny, Eva Chytilova, Jan Kvasnicka, Maja Spiritovic, Barbora Hermankova, Petr Cesak, Marian Rybar, Karel Pavelka, Ladislav Senolt, Herman Mann, Jiri Vencovsky, Michal Vrablik, Michal Tomcik

**Affiliations:** ^1^Institute of Rheumatology, Prague, Czechia; ^2^Department of Rheumatology, 1st Faculty of Medicine, Charles University, Prague, Czechia; ^3^3rd Department of Internal Medicine, General University Hospital and 1st Faculty of Medicine, Charles University, Prague, Czechia; ^4^Department of Physiotherapy, Faculty of Physical Education and Sport, Charles University, Prague, Czechia; ^5^Department of Human Movement Laboratory, Faculty of Physical Education and Sport, Charles University, Prague, Czechia; ^6^Faculty of Mathematics and Physics, Charles University, Prague, Czechia

**Keywords:** atherosclerosis, myositis, inflammation, cardiovascular risk, risk assessment

## Abstract

**Background:**

Idiopathic inflammatory myopathies (IIM) are associated with systemic inflammation, limited mobility, and glucocorticoid therapy, all of which can lead to metabolism disturbances, atherogenesis, and increased cardiovascular (CV) risk. The aim of this study was to assess the CV risk in IIM patients and healthy controls (HC), and its association with disease-specific features.

**Methods:**

Thirty nine patients with IIM (32 females; mean age 56; mean disease duration 4.8 years; dermatomyositis: *n* = 16, polymyositis: *n* = 7, immune-mediated necrotizing myopathy: *n* = 8, anti-synthetase syndrome: *n* = 8) and 39 age-/sex-matched HC (32 females, mean age 56) without rheumatic diseases were included. In both groups, subjects with a history of CV disease (angina pectoris, myocardial infarction, cerebrovascular, and peripheral arterial vascular events) were excluded. Muscle involvement, disease activity, and tissue damage were evaluated (Manual Muscle Test-8, Myositis Intention to Treat Activity Index, Myositis Damage Index). Comorbidities and current treatment were recorded. All participants underwent examinations of carotid intima-media thickness (CIMT), pulse wave velocity (PWV), ankle-brachial index (ABI), and body composition (by densitometry and bioelectric impedance). The risk of fatal CV events was evaluated by the Systematic COronary Risk Evaluation (SCORE, charts for the European population) and its modifications.

**Results:**

Compared to HC, there was no significant difference in IIM patients regarding blood pressure, ABI, PWV, CIMT, and the risk of fatal CV events by SCORE or SCORE2, or subclinical atherosclerosis (CIMT, carotid plaques, ABI, and PWV). The calculated CV risk scores by SCORE, SCORE2, and SCORE multiplied by the coefficient 1.5 (mSCORE) were reclassified according to the results of carotid plaque presence and CIMT; however, none of them was demonstrated to be significantly more accurate. Other significant predictors of CV risk in IIM patients included age, disease duration and activity, systemic inflammation, lipid profile, lean body mass, and blood pressure.

**Conclusions:**

No significant differences in CV risk factors between our IIM patients and HC were observed. However, in IIM, CV risk was associated with age, disease duration, duration of glucocorticoid therapy, lipid profile, and body composition. None of the currently available scoring tools (SCORE, SCORE2, mSCORE) used in this study seems more accurate in estimating CV risk in IIM.

## Introduction

Idiopathic inflammatory myopathies (IIM) are orphan diseases with diverse clinical presentations affecting primarily the skeletal muscle (1). The most prevalent and first recognized subtypes are dermatomyositis (DM), polymyositis (PM) (2, 3), and inclusion body myositis (IBM) (4). Most patients previously diagnosed with PM are now classified as immune-mediated necrotizing myopathy (IMNM) or antisynthetase syndrome (ASS) (5). Despite their various histopathologic and clinical features, all subtypes share similar pathologic mechanisms and involvement of the immune system and inflammation (6).

Inflammation plays a key role in atherogenesis. It has been established that cardiovascular (CV) morbidity and mortality due to exacerbation of atherogenesis is higher in patients with autoimmune diseases than in the general population (7, 8). Moreover, traditional risk factors, such as aging, dyslipidaemia, arterial hypertension, dysregulation of glucose metabolism, and smoking, promotes vascular damage, the formation of atherosclerotic plaques, and ultimately to atherosclerosis (ATS) (9). These traditional risk factors can be attributed to only about 75% of CV manifestations in rheumatic patients (10). Therefore, the inflammatory burden, as a non-traditional risk factor, appears to increase CV risk (11).

Unlike more common rheumatic diseases, few studies have evaluated CV risk in rarer rheumatic diseases like IIM. CV diseases are the leading cause of mortality in IIM patients, who have a 2.24 times higher CV risk (12), and almost four times higher overall mortality compared to the general population (13). An increased risk of myocardial infarction (MI) has been observed especially during the first years of IIM (14) due to accelerated ATS (15).

In the general population, Systemic COronary Risk Evaluation (SCORE) (16), or the recently validated SCORE2 (17) are the most widely used CV risk scoring systems in the European populations. Given the increased CV risk associated with inflammatory rheumatic diseases, European Alliance of Associations for Rheumatology (EULAR) recommended modifying the scoring systems for patients with inflammatory arthropathy, such as rheumatoid arthritis (RA), psoriatic arthritis (PsA), and ankylosing spondylitis (AS) by multiplying SCORE by the coefficient 1.5 (mSCORE) (18). However, there is no specific recommended scoring system to estimate CV risk in IIM or other orphan rheumatic diseases.

Moreover, according to a large Atherosclerosis Risk in Communities (ARIC) study in the general population, the measurement of carotid intima-media thickness (CIMT) and plaque detection by B-mode ultrasound examination can significantly improve CV risk prediction (19). The ultrasound examination of carotid arteries, together with other non-invasive methods, such as ankle-brachial index (ABI) and carotid-femoral pulse wave velocity (cf-PWV), are the standard non-invasive methods widely used for assessing subclinical ATS (11, 20, 21).

This cross-sectional pilot study aimed to evaluate CV risk in IIM patients, compare it to healthy controls, and assess factors contributing to CV risk in IIM patients.

## Materials and Methods

### Patients and Healthy Controls

This is a cross-sectional, observational, prospective study on CV risk in patients with IIM compared to age-/sex-matched HC. In total, 39 patients with IIM who fulfilled the classification criteria for adult IIM (22), were consecutively recruited from May 2018 to April 2021 at the Institute of Rheumatology in Prague (IoRP). Inclusion criteria were: aged 18 years and older, regularly followed up by the attending rheumatologist, and treated with standard-of-care therapy. Exclusion criteria included other rheumatic disease, active neoplasia, chronic infection, and a history of manifested ATS and CV disease (i.e., angina pectoris, myocardial infarction, cerebrovascular events including stroke or transient ischemic attack, peripheral artery disease, or peripheral embolization). IIM patients were excluded in the case of severe, life-threatening diseases, severe lung involvement requiring continuous oxygen therapy, or disability to undergo all examinations. In total, 39 healthy controls (HC) matched by age and sex, without any rheumatic disease, chronic diseases including chronic infectious disease, active neoplasia, or a history of manifested atherosclerosis or CV disease, were enrolled from the Healthy Control Registry of IoRP, consisting of employees, their relatives and acquaintances using the snowball method. The detailed recruitment process of IIM patients and HC is presented in Supplementary Figure 1. All participants signed an informed consent prior to inclusion in the study, which was approved by the local Ethics Board of IoRP. All examinations were performed according to the relevant regulations and guidelines.

### Baseline Characteristics

In IIM patients, disease-specific features were assessed by an experienced rheumatologist according to international guidelines (23). Disease activity and tissue damage were evaluated by the Myositis Intention-to-Treat Activity Index (MITAX) and the Myositis Damage Index (MDI) (24), respectively, muscle involvement by the Manual Muscle Testing (MMT)-8 (25), and comorbidities were recorded from the medical documentation. In all participants, traditional risk factors were recorded from medical documentation and a questionnaire on health status. All participants filled out the Patient Reported Outcomes (PRO) questionnaires on functional status (Health Assessment Questionnaire, HAQ) (25, 26), quality of life (Medical Outcomes Study 36-item Short Form Health Survey, SF-36) (25, 27), fatigue [Fatigue Impact Scale, FIS (28) and Multidimensional Assessment of Fatigue Scale, MAF (29)], physical activity (Human Activity Profile, HAP) (30), and depression (Beck's Depression Inventory-II, BDI-II) (31). Further details on the PRO questionnaires used and the validated Czech versions can be found in our recently published studies (32, 33). Additionally, current therapy with glucocorticoids (GC) and immunosuppressive drugs was documented; total cumulative dose of GC (prednisolone equivalent dose) and total exposure time to GC were calculated based on patients' medical documentation.

### Laboratory Methods

Blood samples were obtained after 8 h of fasting. Routine biochemical assessment was performed using the Beckman CoulterAU 680 analyzer (Beckman Coulter, USA) for C-reactive protein (CRP), fasting glucose, fasting plasma concentrations of insulin, C-peptide, calcitriol (1,25-dihydrocholecalciferol) and markers of lipid metabolism: total cholesterol (TC), low-density lipoprotein cholesterol (LDL), high-density lipoprotein cholesterol (HDL), triglycerides (TAG), lipoprotein(a), apolipoprotein-A1 (apo-A), and apolipoprotein-B (apo-B). Atherogenic index of plasma (AI) was calculated as log (TAG/HDL) (34). All participants underwent a glucose tolerance test, except for those previously diagnosed with diabetes mellitus. In IIM patients, laboratory tests also included erythrocyte sedimentation rate (ESR, according to the Fahreus and Westergren method), muscle damage markers: creatine kinase (CK), myoglobin, and lactate dehydrogenase (LDH), antinuclear antibodies (ANA, using indirect immunofluorescence on HEP2 cells), and myositis–specific (MSA) and myositis-associated antibodies (MAA) by the Myositis Line Immunoassay (Human Diagnostica, Wiesbaden, Germany) and Myositis Westernblot (Euroimmun, Lübeck, Germany). Blood samples were analyzed for plasma levels of selected cytokines and chemokines using the Bio-Plex Pro^TM^ Human Cytokine 27-plex Assay (BIO-RAD, California, USA) as previously described (33): interleukin (IL)-1β, IL-1Ra, IL-2, IL-4, IL-5, IL-6, IL-7, IL-8, IL-9, IL-10, eotaxin, interferon gamma-induced protein (IP)-10 (CXCL10), monocyte chemoattractant protein (MCP)-1 (CCL2), macrophage inflammatory proteins (MIP)-1α (CCL3), and MIP-1β (CCL4), platelet-derived growth factor (PDGF)-bb, regulated on activation/normal T cell expressed and secreted (RANTES, CCL5), and tumor necrosis factor (TNF).

### Assessment of Body Composition

Body-mass index (BMI) was calculated, and body composition was assessed using bioelectrical impedance analysis (BIA) and dual-energy X-ray absorptiometry (DXA). BIA was performed using a multi-frequency bioelectrical impedance analyzer (BIA-2000M, Data Input GmbH, Pöcking, Germany), according to the standardized protocol (35) in the morning after 8 h of fasting, as described in a previous study (33). Densitometry was performed using Lunar-iDXA (GE Healthcare, Milwaukee, WI, USA) according to the manufacturer's protocol. The basic parameters evaluated in both methods were body fat (BF)%, lean body mass (LBM), and fat-free mass (FFM). Other parameters of interest included bone mineral content (BMC), android/gynoid fat ratio, resting metabolic rate (RMR), Relative Skeletal Muscle Index (RSMI), estimated visceral adipose tissue volume and mass for DXA, and total body water (TBW), intracellular water (ICW), extracellular water (ECW), extracellular mass (ECM), body cell mass (BCM), ECM/BCM, and BCM/FFM for BIA, as described elsewhere (33).

### Assessment of Subclinical Atherosclerosis

All participants underwent an ultrasound examination of carotid arteries to evaluate the carotid intima-media thickness (CIMT), the presence of plaque (carotid artery disease), pulse wave velocity (PWV), and ankle brachial index (ABI) on the same day or within 2 weeks after blood sample collection and body composition analysis. Measurements were performed by two trained cardiologists (one for CIMT, one for PWV) and one experienced cardiology nurse (for ABI), blinded to the group allocation. Patients were educated to fast 2 h prior to examination and strictly avoid drinking coffee, tea or alcohol 12 h prior to examination.

#### Carotid Intima-Media Thickness

CIMT was assessed by carotid ultrasound (Vivid 9 ultrasound system, GE Healthcare, Chicago, IL, USA) using a 15-MHz linear array transducer ML6-15-D over a 1 cm segment of the common carotid artery, 1 to 2 cm proximal to the carotid bifurcation as described elsewhere (36). The mean of six measurements in both carotid arteries (3 on each side) was documented. We used two criteria to classify pathologic CIMT values: (i) over the 90th percentile of the corresponding age and sex groups (37), and (ii) CIMT >0.9 mm (38).

#### Carotid Plaques

Carotid atherosclerotic plaques were screened for in common, internal and external carotids. The recent recommendations from the American Society of Echocardiography define carotid plaque as any focal thickening encroaching into the lumen of any segment of the carotid artery (protuberant-type plaque) or thickening of artery wall ≥1.5 mm as the cutoff value of CIMT (diffuse-type plaque) (39).

#### Ankle Brachial Index

The ankle brachial index was examined by Doppler ultrasound (Doplex mini D900, Huntleigh Healthcare, Cardiff, Wales, United Kingdom) on the posterior tibial artery and the dorsalis pedis artery of each foot, and blood pressure was meas-ured by an automatic sphygmomanom-eter (M3 Comfort, Omron, Kyoto, Japan), with subjects in the supine position after 5 min of rest; the average of three consecu-tive measurements was recorded. ABI values <0.9 were considered pathologic (0.8–0.9 incipient arterial disease, 0.5–0.8 moderate arterial disease, <0.5 severe arterial disease) (40).

#### Pulse Wave Velocity

Aortal stiffness was measured by the carotid-femoral pulse wave velocity (cf-PWV) using SphygmoCor CV Management System (CvMS) software version 9 (AtCor Medical, Sydney, Australia) according to protocol (41, 42). The mean of three measurements was documented. Values were estimated according to the Reference Value for Arterial Stiffness Collaboration (2010) standardized for the normal (no additional CV risk factor) and reference (presenting CV risk factor) population, categorized according to age and blood pressure: normal values range from 6.2 m/s (age <30) to 10.9 m/s (age >70) (43).

### Evaluation of CV Risk by Scoring Systems

CV risk was evaluated using scoring systems estimating the 10-year risk of fatal CV events (SCORE), and fatal and non-fatal CV events (SCORE2). We applied SCORE (16), SCORE2 (17), and mSCORE (18) in IIM, and SCORE and SCORE2 in HC. SCORE was calculated according to the appropriate chart for the European population. SCORE2 was calculated using the online calculator (https://u-prevent.nl/calculators) for high-risk population (Czech Republic). mSCORE was SCORE multiplied by 1.5 (18).

### Assessment of CV Risk According to the Cardiovascular and Ultrasound Examination

All participants were divided into three categories of overall CV risk based on the scores and the findings on the carotid ultrasound examination: (i) the low-risk category included individuals with SCORE or mSCORE with a 10-year risk of fatal CV events <5%, SCORE2 with a 10-year risk of fatal and non-fatal CV events <2.5%, 5% or 7.5% (according to age), and normal values of CIMT and no plaque formation was detected; (ii) the intermediate-risk category included individuals with a calculated CV risk of fatal CV events between 5 and 10% for SCORE or mSCORE and a risk of fatal and non-fatal CV events 2.5–7.5, 5–10, or 7.5–15% (according to age) for SCORE2, and normal CIMT and a maximum of one plaque <1.9 mm; (iii) the high-risk category included individuals with a calculated CV risk ≥10% for SCORE or mSCORE and ≥7.5%, ≥10%, or ≥15% (according to age) for SCORE2, or pathologic CIMT or one plaque >1.9 mm or >1 carotid plaques (16–19, 44).

### Statistical Analysis

Statistical analysis was performed using STATISTICA 12 (StatSoft CR s.r.o., Prague, Czech Republic). Continuous variables were reported as means with standard deviation (SD), and the two-sample *t*-test was used for a simple comparison between groups in the univariate analysis. Categorical variables were reported as percentages, and Pearson's chi-squared test was used to compare groups in the univariate analysis. The relationships between two parameters were evaluated by the Pearson's correlation coefficient. *P*-values <0.05 were considered statistically significant. Variables with *p* <0.25 from univariate analyses were taken into a multivariate logistic regression analysis to determine an adjusted influence of variables on the outcome. A *p*-value of < 0.05 was then used in the final model. The graphs were created by Excel 2016 (v 16.0, Redmond, Washington, USA). Data are presented as mean ± SD or median (interquartile range).

## Results

### Clinical Characteristics

We have included 39 patients with idiopathic inflammatory myopathies [32 (82%) females, 7 (18%) males, mean ± SD age 56.0 ± 11.0 years], and 39 age-/sex-matched healthy controls [32 (82%) females, 7 (18%) males, mean age 55.9 ± 11.2 years]. IIM patients had the following subtypes: DM (*n* = 16; 41%), PM (*n* = 7; 18%) IMNM (*n* = 8; 20.5%) and ASS (*n* = 8; 20.5%). Median disease duration was 4.8 years, and disease activity was predominantly mild (MITAX 0.13) and disease damage low (MDI 0.05). The most common manifestation was muscle weakness (88%) and interstitial lung disease (ILD) (41%). ILD was defined as interstitial lung fibrosis or alveolitis based on high-resolution computed tomography and pulmonary consultation excluding other etiology of lung involvement (45). Most of the patients were treated with GC (92%). Methotrexate (MTX) (28%) and azathioprine (AZA) (21%) prevailed among conventional synthetic disease-modifying anti-rheumatic drugs (csDMARDs). The mean cumulative dose of GC (prednisolone equivalent dose) during the course of the disease was 13,052 mg and the mean duration of GC exposure was 3.9 years. The baseline characteristics of both groups are shown in Table 1.

**Table 1:** Clinical and demographic characteristics of IIM patients and healthy controls.

**Parameter**	**IIM (*n* = 39)**	**HC (*n* = 39)**
Gender, *n* (%): female/male	32 (82)/7 (18)	32 (82)/7 (18)
Age (years)	56.0 (47.7–64.1)	56.0 (48.3–64.2)
BMI (kg/m^2^)	25.9 (23.2–31.1)	27.5 (23.9–31.7)
**Clinical features**		
Disease subtype, *n* (%): DM/PM/IMNM/ASS	16 (41)/7 (18)/8 (20.5)/8 (20.5)	
Disease duration (years)	4.84 (1.96–8.83)	
Disease activity (MITAX)	0.13 (0.06–0.29)	
Disease damage (MDI)	0.05 (0.03–0.08)	
Muscle strength (MMT-8)	64 (54–70)	
IIM-associated clinical manifestations, *n* (%): MW/OD/SR/MH/RP/A/ILD/CI	35 (88)/7 (18)/5 (13)/8 (21)/10 (26)/5 (13)/16 (41)/3 (8)	
**Laboratory features**		
CRP (mg/L)	3.0 (1.4–5.0)	
ESR (mm/h)	13 (8–25)	
CK (μkat/L)	3.0 (1.3–9.4)	
LD (μkat/L)	3.7 (3.4–5.2)	
Myoglobin (μg/L)	93.6 (60.4–250.2)	
Autoantibodies (positive), *n* (%):		
ANA/Mi-2/TIF-1γ/MDA-5/SAE/NXP-2/SRP/Jo-1/PM-Scl/	24 (62)/3 (8)/3 (8)/0 (0)/0 (0)/0 (0)/3 (8)/10 (26)/5 (13)/	
RNP/Ku/Ro-52/Ro-60/HMGCR	5 (13)/0 (0)/12 (31)/7 (18)/2 (5)	
**Treatment**		
Current dose of GC—prednisolone equivalent dose (mg/day)	6.9 (2.5–16.3)	
Cumulative dose of GC—prednisolone equivalent dose (mg)	13,052 (7,921–29,055)	
GC exposure (years)	3.9 (0.9–8.6)	
Current treatment, *n* (%):		
GC/MTX/AZA/CSA/CPA/LEF/MMF	36 (92)/11 (28)/8 (21)/5 (13)/2 (5)/2 (5)/1 (3)	

*Data are presented as median (inter-quartile range) unless stated otherwise. A, arthritis; ANA, antinuclear antibodies; ASS, antisynthetase syndrome; AZA, azathioprine; BMI, body-mass index; CI, cardiac involvement; CK, creatine kinase; CPA, cyclophosphamide; CRP, C-reactive protein; CSA, cyclosporin A; DM, dermatomyositis; ESR, erythrocyte sedimentation rate; GC, glucocorticoids; HC, healthy controls; IIM, idiopathic inflammatory myopathies; ILD, interstitial lung disease; IMNM, immune-mediated necrotising myopathy; Jo-1, anti-histidyl-tRNA synthetase; Ku, anti-Ku (against the nuclear DNA-protein kinase subunit); LD, lactate dehydrogenase; LEF, leflunomide; MDA-5, anti-antigen associated with melanoma differentiation; MMF, mycophenolate mofetil; MH, mechanic's hands; Mi-2, anti-nuclear helicase 218/240 kDa; MITAX, Myositis Intention to Treat Activity Index; MMT-8, manual Muscle Testing-8; MTX, methotrexate; MW, muscle weakness; NXP2, anti-nuclear matrix protein; OD, oesophageal motility disorder; PM, polymyositis; PM-Scl, anti-Pm-Scl (anti-core complex 11–16 proteins); RNP, anti-ribonucleoprotein; Ro, anti-Ro (52/60 kDa, against cytoplasmic RNA and associated peptides); RP, Raynaud's phenomenon; SAE, anti-SUMO1 (small ubiquitin-like activating enzyme); SR, skin rash; SRP, anti-signal recognition particles; TIF1, anti-transcription factor-1*.

### Traditional Risk Factors

The prevalence of traditional risk factors was not significantly different between IIM and HC. There was a trend to a higher occurrence of diabetes in IIM (15%) compared to HC (5%) (*p* = 0.136). The presence of other traditional CV risk factors was comparable in both cohorts. The only notable difference was in current alcohol intake, where IIM patients reported no alcohol consumption, while the majority of HC consumed alcohol (64%) (*p* < 0.001) (Table 2).

**Table 2:** Comorbidities and traditional cardiovascular risk factors in IIM and HC.

**Traditional cardiovascular risk factor**	**IIM (*n* = 39)**	**HC (*n* = 39)**	***p*** **-value**
Gender, *n* (%): female/male	32 (82)/7 (18)	32 (82)/7 (18)	–
BMI (kg/m^2^); median (IQR)	25.9 (23.2–31.1)	27.5 (23.9–31.7)	0.422
Arterial hypertension (treated or untreated); *n* (%)	18 (46.2)	21 (53.8)	0.497
Antihypertensive treatment; *n* (%)	11 (28.2)	12 (30.8)	0.804
Dyslipidaemia (treated or untreated); *n* (%)	28 (71.8)	24 (61.5)	0.337
Hypolipidemics (statins, fibrates); *n* (%)	2 (5.1)	3 (7.7)	0.644
Prediabetes; *n* (%)	11 (28.2)	10 (25.6)	0.799
Diabetes; *n* (%)	6 (15.4)	2 (5.1)	0.136
Insulin treatment; *n* (%)	2 (5.1)	0 (0)	0.152
Peroral antidiabetic drugs; *n* (%)	2 (5.1)	2 (5.1)	1.000
Smoking; *n* (%)	3 (7.7)	5 (12.8)	0.456
Family history of cardiovascular diseases; *n* (%)	6 (15.4)	9 (23.1)	0.389
Alcohol (regular drinking); *n* (%)	0 (0)	25 (64.1)	**<0.001**

*Statistically significant differences (p < 0.05) are highlighted in bold. BMI, body-mass index; HC, healthy controls; IIM, idiopathic inflammatory myopathies; IQR, inter-quartile range*.

### Comparison of the CV Risk Between IIM Patients and Healthy Controls

The results of non-invasive CV examinations, i.e., carotid plaques (presence, total count, and total sum of thickness of plaques in each individual), CIMT, ABI, and PWV, were compared between IIM and HC using the two-sample *t*-test, and the chi-square test for the presence of plaques. Only PWV was significantly different between the two cohorts. The mean PWV was significantly increased in IIM compared to HC (7.98 ± 2.12 vs. 5.96 ± 4.01 m/s, *p* = 0.015). However, the percentage of pathologic results of PWV was comparable in both cohorts with only a weak trend toward higher values in IIM (*p* = 0.176). On the other hand, the prevalence of pathologic CIMT and ABI were comparable in both groups (*p* = 0.498, *p* = 0.411, respectively). Similarly, the calculated SCORE and SCORE2 did not significantly differ between both cohorts (*p* = 0.847, *p* = 0.519, respectively).

To exclude the impact of traditional CV risk factors, all parameters of CV ultrasound examination, as well as SCORE and SCORE2, were adjusted for the traditional CV risk factors (Table 2). Adjustment for age and gender was unnecessary, since both cohorts were matched for age and sex. Initially, the chi-square test was used to assess multicollinearity. Due to the identified collinearity between arterial hypertension and antihypertensive therapy, between diabetes and insulin therapy, and between antihypertensive and insulin therapy, the latter parameters were excluded from further analysis. Subsequently, univariate analysis was performed, and significant variables (*p* <0.250) were analyzed in a multivariate model. After the adjustment and subsequent analysis, no significantly different factors arose. Moreover, the significance of PWV values was lost. In short, after adjusting for traditional CV risk factors, no significant difference was observed between the IIM and HC cohorts regarding the findings from CV examinations (carotid plaques, CIMT, ABI and PWV) and the CV risk estimated by SCORE and SCORE2 (Table 3).

**Table 3:** Subclinical atherosclerosis (ultrasound and cardiovascular examination) and cardiovascular risk (SCORE, SCORE2) in IIM and HC.

**Parameter**	**IIM (*n* = 39)**	**HC (*n* = 39)**	***p*** **-value**
Coronary artery disease (carotid plaques); *n* (%)	15 (38.5)	11 (28.2)	0.337
Carotid plaques total count	1.13 (1.64)	0.87 (1.51)	0.475
Carotid plaques total thickness (mm)	1.07 (1.07)	0.86 (1.57)	0.572
CIMT (mm)	0.72 (0.15)	0.69 (0.15)	0.328
ABI	0.988 (0.158)	1.019 (0.158)	0.393
PWV (m/s)	7.98 (2.12)	5.96 (4.01)	**0.015**
Pathologic CIMT (>0.9 mm); *n* (%)	6 (15.4)	4 (10.3)	0.498
Pathologic ABI (<0.9); *n* (%)	7 (17.9)	10 (25.6)	0.411
Pathologic PWV; *n* (%)	7 (17.9)	3 (7.7)	0.176
SCORE	3.18 (3.12)	3.31 (2.74)	0.847
SCORE2	11.3 (15.75)	9.33 (10.68)	0.519

*Data are presented as mean (standard deviation) unless stated otherwise. Statistically significant differences (p < 0.05) are highlighted in bold. ABI, ankle-brachial index; CIMT, carotid intima-media thickness; HC, healthy controls; IIM, idiopathic inflammatory myopathies; PWV, pulse wave velocity; SCORE, Systematic COronary Risk Evaluation*.

### Cardiovascular Risk Categories

To evaluate the overall CV risk in both cohorts, we divided the individuals into four categories based on the level of risk calculated in (i) SCORE (CVR-SCORE), (ii) SCORE2 (CVR-SCORE2), (iii) mSCORE (CVR-mSCORE), and (iv) carotid ultrasound findings including CIMT, presence of carotid plaque, and plaque thickness (CVR-US). Each category was divided into three levels of risk: Level 1 = low risk, defined as a 10-year risk of fatal CV events of <5% (SCORE, mSCORE) or fatal and non-fatal CV events of <2.5, 5 or 7.5% (according to age, SCORE2) for categories i–iii, and as CIMT <0.9 mm and no carotid plaques for category iv; Level 2 = intermediate risk, defined as a 10-year risk of fatal CV events of 5–10% (SCORE, mSCORE) or fatal and non-fatal CV events of 2.5–7.5, 5–10, or 7.5–15% (according to age, SCORE2) for categories i–iii, and as CIMT 0.9–1 mm or 1 carotid plaque <1.9 mm for category iv; Level 3 = high risk, defined as a 10-year risk of fatal CV events of ≥10% or fatal and non-fatal CV events of ≥7.5, ≥10 or ≥15% (according to age, SCORE2) for categories i–iii, and as CIMT >1 mm or 1 carotid plaque >1.9 mm or >1 carotid plaque (total plaque thickness>1.9 mm) for category iv (Table 4). The mean level of each category in both cohorts was calculated, and compared between IIM and HC using two-sample *t*-test. No significant difference was found (*p* > 0.05 for all), suggesting that IIM patients have a comparable CV risk to HC, evaluated both by SCORE, SCORE2, and carotid ultrasound examination (Table 5). mSCORE was not included in this analysis, as it has been proposed for inflammatory arthropathies, and herein calculated for IIM only.

**Table 4:** Definition of the cardiovascular risk categories and levels based on the CV risk score (SCORE, SCORE2, mSCORE) and carotid ultrasound examination (CIMT, and plaque detection).

**Cardiovascular risk category**	**Based on**	**Level**	**Definition**
CVR-SCORE	Calculated SCORE	1	<5%
		2	5–10%
		3	>10%
CVR-SCORE2	Calculated SCORE2	1	<2.5% (for <50 years); <5% (for 50–69 years); <7.5% (for ≥70 years)
		2	2.5-7.5% (for <50 years); 5–10% (for 50–69 years); 7.5–15% (for ≥70 years)
		3	≥7.5% (for <50 years); ≥10% (for 50–69 years); ≥15% (for ≥70 years)
CVR-mSCORE	Calculated mSCORE	1	<5%
		2	5–10%
		3	>10%
CVR-US	Carotid ultrasound	1	CIMT <0.9 mm AND no carotid plaques
		2	CIMT 0.9–1 mm OR 1 carotid plaque <1.9 mm
		3	CIMT > 1 mm OR 1 carotid plaque >1.9 mm OR >1 carotid plaque <1.9 mm

*1, low; 2, intermediate; 3, high risk; CIMT, carotid intima-media thickness; CVR, cardiovascular risk; mSCORE, modified Systematic COronary Risk Evaluation; SCORE, Systematic COronary Risk Evaluation; US, carotid ultrasound examination*.

**Table 5:** Comparison of the cardiovascular risk levels in the cardiovascular risk categories between IIM and HC.

**Cardiovascular risk category**	**CVR level IIM** **(*n* = 39)**	**CVR level HC** **(*n* = 39)**	***p*** **-value**
CVR-SCORE	1.33 (0.58)	1.38 (0.54)	0.687
CVR-SCORE2	1.79 (0.83)	1.97 (0.78)	0.297
CVR-US	1.72 (0.94)	1.56 (0.91)	0.467

*Data are presented as mean (standard deviation). CVR, Cardiovascular risk; CVR-SCORE, Cardiovascular risk estimated according to the calculated SCORE; CVR-SCORE2, Cardiovascular risk estimated according to the calculated SCORE2; CVR-US, Cardiovascular risk estimated according to the carotid ultrasound examination (total plaque count, plaque thickness, carotid intima-media thickness); IIM, idiopathic inflammatory myopathies; HC, healthy controls; SCORE, Systematic COronary Risk Evaluation*.

### Change in CV Risk Categorization

To verify which scoring system (SCORE, SCORE2, or mSCORE,) corresponds best to the CV risk estimated by carotid ultrasound examination (CIMT, plaque count, and thickness), we reclassified every individual from the CVR-SCORE, CVR-SCORE2, or CVR-mSCORE category according to the findings on carotid ultrasound examination: the CV risk level increased in the case of plaques or high CIMT; on the contrary, individuals in the intermediate-risk or high-risk level based on a calculated scoring system, with normal CIMT and no plaque, remained at the same CV risk level (Tables 6, 7, Supplementary Figures 2A–E). Based on SCORE, 28 (72%) IIM patients were classified as low risk, 9 (23%) as intermediate risk, and 2 (5%) as high risk. After comparing SCORE and ultrasound findings, 14 (36%) patients in total were reclassified to a higher risk level (Table 6, Supplementary Figure 2A). When evaluating SCORE2, 18 (46%) IIM patients were originally classified as low risk, 11 (28%) as intermediate risk, and 10 (26%) as high risk. After carotid ultrasound examination, 6 (15%) in total were reclassified to a higher risk level (Table 6, Supplementary Figure 2B). Finally, using mSCORE, 26 (67%) of IIM patients were at low risk, 5 (13%) at intermediate risk, and 8 (20%) at high risk, and subsequently, 9 (23%) in total were reclassified to a higher CV risk level due to the findings of carotid ultrasound (Table 6, Supplementary Figure 2C). Based on the total number of reclassified IIM patients, SCORE seemed to underestimate CV risk more than SCORE2 and mSCORE, whereas SCORE2 was the most accurate when compared to carotid ultrasound findings. Nevertheless, there were no statistically significant differences between the three scoring tools in underestimating or overestimating CV risk (Table 6).

**Table 6:** Reclassification of the original cardiovascular risk category in IIM based on SCORE, SCORE2, and mSCORE to the cardiovascular risk category based on subclinical atherosclerosis markers on carotid ultrasound examination.

**CVR scoring system**	**Original CVR category**	**Patients,** ***n* (%)**	**New CVR category**	**Better/same/** **worse**	***n***	**%**	**Total *n* (%) of reclassified to a higher CV risk level**
CVR-SCORE	1	28 (71.8)	1	Same	20	51.3	Total 14 (35.9)
			2	Worse	2	5.1	
			3	Worse	6	15.4	
	2	9 (23.1)	2	Same	3	7.7	
			3	Worse	6	15.4	
	3	2 (5.1)	3	Same	2	5.1	
CVR-SCORE2	1	18 (46.1)	1	Same	17	43.6	Total 6 (15.4)
			2	Worse	0	0.0	
			3	Worse	1	2.6	
	2	11 (28.2)	2	Same	6	15.4	
			3	Worse	5	12.8	
	3	10 (25.6)	3	Same	10	25.6	
CVR-mSCORE	1	26 (66.7)	1	Same	20	51.3	Total 9 (23.1)
			2	Worse	1	2.6	
			3	Worse	5	12.8	
	2	5 (12.8)	2	Same	2	5.1	
			3	Worse	3	7.7	
	3	8 (20.5)	3	Same	8	20.5	

*CVR, Cardiovascular risk; SCORE – Systematic COronary Risk Evaluation; CVR-SCORE, Cardiovascular risk estimated according to the calculated SCORE; CVR-SCORE2, Cardiovascular risk estimated according to the calculated SCORE2; CVR-US, Cardiovascular risk estimated according to the carotid ultrasound examination (total plaque count, plaque thickness, carotid intima-media thickness)*.

**Table 7:** Reclassification of the original cardiovascular risk category in HC based on SCORE, SCORE2, and mSCORE to the cardiovascular risk category based subclinical atherosclerosis markers on carotid ultrasound examination.

**CVR scoring system**	**Original CVR category**	**Patients,** ***n* (%)**	**New CVR category**	**Better/same/** **worse**	***n***	**%**	**Total *n* (%) of reclassified to a higher CV risk level**
CVR-SCORE	1	25 (64)	1	Same	22	56.4	Total 11 (28.1)
			2	Worse	0	0.0	
			3	Worse	3	7.7	
	2	13 (33)	2	Same	5	12.8	
			3	Worse	8	20.5	
	3	1 (3)	3	Same	1	2.56	
CVR-SCORE2	1	12 (31)	1	Same	12	30.8	Total 2 (5.1)
			2	Worse	0	0.0	
			3	Worse	0	0.0	
	2	16 (41)	2	Same	13	33.3	
			3	Worse	2	5.1	
	3	11 (28)	3	Same	12	30.8	

*CVR, Cardiovascular risk; SCORE, Systematic COronary Risk Evaluation; CVR-SCORE, Cardiovascular risk estimated according to the calculated SCORE; CVR-SCORE2, Cardiovascular risk estimated according to the calculated SCORE2; CVR-US, Cardiovascular risk estimated according to the carotid ultrasound examination (total plaque count, plaque thickness, carotid intima-media thickness)*.

To compare the reliability of the CV risk tools in IIM and the general population, we also performed the same reclassification in our HC cohort (Table 7, Supplementary Figures 2D,E). In HC evaluated by SCORE, 25 (64%) individuals were classified as low risk, 13 (33%) as intermediate risk, and 1 (3%) as high risk. Overall, 11 (28%) individuals were reclassified to a higher CV risk level after ultrasound examination (Table 7, Supplementary Figure 2D). Regarding SCORE2 in HC, the CV risk was low in 12 (31%) individuals, intermediate in 16 (41%), and high in 11 (28%). After reassessment, the CV risk level changed in 2 (5%) to a higher CV risk level (Table 7, Supplementary Figure 2E).

Although the above-mentioned numbers and differences (Tables 6, 7, Supplementary Figures 2A–E) could indicate that some scoring systems could estimate CV risk more accurately than others, this assumption was not confirmed in IIM patients by the subsequent statistical analysis. We compared the percentages of congruency and disparity between each pair (CVR-SCORE vs. CVR-US, CVR-SCORE2 vs. CVR-US, and CVR-mSCORE vs. CVR-US) using chi-square test. There was no significant difference in the percentage of changes, or the ability to estimate CV risk by any method used in IIM patients (*p* = 0.106). On the contrary, in HC, we observed significantly higher total percentage of reclassified HC from SCORE (28%) compared to SCORE2 (5%) (*p* = 0.006 comparing CVR-SCORE vs. CVR-US with CVR-SCORE2 vs. CVR-US). Therefore, we have observed high accuracy of SCORE2, while SCORE underestimated CV risk in healthy controls.

### Association of the CV Risk and Disease-Specific Features in IIM Patients

Next, we analyzed the potential associations between disease-specific features, selected based on a priori clinical judgment, and CV risk or pathologic findings from carotid ultrasound and CV examination. Statistically significant correlations (Pearson's correlation coefficient) from the univariate analysis were selected and subsequently analyzed in a multivariate model. The univariate analysis demonstrated that age and mean arterial pressure were the most significant parameters correlated positively with the calculated CV risk by SCORE, SCORE2, and mSCORE. This is not surprising since all SCORE calculators include age and blood pressure. Furthermore, the univariate analysis revealed other significant associations of lipid profile, body composition, disease activity and serum levels of cytokines/chemokines (IL-9 and IP-10) with CIMT, carotid plaques, and overall CV risk estimated by US. As presumed, we also observed an association between CV risk (estimated by scoring systems and US examination) and CV examination findings. The significant correlations from the univariate analysis are shown in Table 8. Furthermore, categorical variables were also tested for significant associations with CV risk and the findings from CV examination. We included traditional risk factors and their treatment (Table 2), the presence of the most prevalent autoantibodies (ANA, anti-Jo-1, and anti-Ro-52), clinical features [Raynaud's phenomenon (RP), mechanic's hands (MH) and ILD], and pharmacotherapy (GC, MTX, and AZA) (Table 9).

**Table 8:** Association of cardiovascular risk factors and subclinical atherosclerosis markers with disease-specific features, body composition parameters, and traditional risk factors in IIM, based on bivariate correlations.

**Correlated parameters**	**Disease features, CV risk factors, body composition**	**Pearson's r**	***p*** **-value**
CIMT	Age	0.560	0.004
ABI	IL-9	0.503	0.040
	TC	0.603	0.010
	HDL	0.531	0.028
	Apo-A	0.488	0.047
PWV	Age	0.684	0.002
	MITAX	−0.575	0.016
	ESR	0.574	0.016
	CRP	0.522	0.031
	IP-10	0.623	0.008
	MAP	0.804	<0.001
Carotid plaques (total count)	Disease duration	0.574	0.016
	MITAX	−0.494	0.044
	LBM-DXA	−0.508	0.037
	ECM/BCM	0.613	0.009
	BMR-DXA	−0.545	0.024
	C-peptide	−0.549	0.022
Carotid plaques	Disease duration	0.517	0.034
(maximum thickness)	MITAX	−0.568	0.017
	LBM-DXA	−0.490	0.046
	ECM/BCM	0.523	0.031
	BMR-DXA	−0.516	0.034
	IP-10	0.538	0.026
CVR-SCORE	Age	0.684	0.002
	apo-B	0.534	0.024
	AI	0.500	0.041
	MAP	0.738	0.001
CVR-SCORE2	Age	0.823	<0.001
	AI	0.546	0.024
	MAP	0.736	0.001
CVR-mSCORE	Age	0.775	<0.001
	MAP	0.716	0.001
CVR-US	MITAX	−0.582	0.014
	Phase angle	−0.498	0.042
	ECM/BCM	0.500	0.041
	C-peptide	−0.524	0.031
PWV	SCORE	0.675	0.003
	SCORE2	0.597	0.011
	mSCORE	0.675	0.003
	c. plaques-total count	0.516	0.034
	c. plaques—max thickness	0.643	0.005
	CVR-US	0.538	0.026
CIMT	SCORE2	0.767	<0.001

*ABI, ankle brachial index; AI, atherogenic index of plasma; apo-A, apolipoprotein-A; BMR-DXA, basal metabolic rate measured by dual energy X-ray absorptiometry; CIMT, carotid intima-media thickness; CRP, C-reactive protein; CV, cardiovascular; CVR, cardiovascular risk; CVR-mSCORE, cardiovascular risk estimated according to the calculated mSCORE; CVR-SCORE, cardiovascular risk estimated according to the calculated SCORE; CVR-SCORE2, cardiovascular risk estimated according to the calculated SCORE2; CVR-US, cardiovascular risk based on carotid ultrasound examination (plaques, CIMT); ECM/BCM, extracellular mass/body cell mass ratio; ESR, erythrocyte sedimentation rate; HDL, high-density lipoprotein; IL-9, interleukin-9; IP-10, interferon-gama-induced protein 10 (CXCL10); LBM-DXA, lean body mass measured by dual energy X-ray absorptiometry; MAP, mean arterial pressure; MITAX, Myositis Intention to Treat Activity Index; PWV, pulse wave velocity; SCORE, Systematic COronary Risk Evaluation; TC, total cholesterol; US, ultrasound (examination)*.

**Table 9:** Significant differences in subclinical atherosclerosis markers and cardiovascular (CV) risk scores based on presence or absence of traditional CV risk factors and their treatment and selected autoantibodies.

**Parameters**	**CV risk factors and their**		***p*** **-value**
	**treatment, antibodies**		
	**Arterial hypertension (** ***n*** **)**		
	**Present (18)**	**Absent (21)**	
SCORE	4.667 (3.36)	1.905 (2.234)	0.004
SCORE2	17.333 (18.846)	6.143 (10.442)	0.025
mSCORE	7.000 (5.093)	2.857 (3.351)	0.004
Carotid plaques (total count)	1.778 (1.896)	0.571 (1.165)	0.020
Carotid plaques (max. thickness)	0.967 (0.944)	0.400 (0.771)	0.046
PWV	8.753 (1.716)	7.207 (2.249)	0.043
CVR-SCORE	1.556 (0.705)	1.143 (0.359)	0.024
CVR-SCORE2	2.298 (0.752)	1.381 (0.669)	0.005
CVR-mSCORE	1.833 (0.924)	1.286 (0.645)	0.036
CVR-US	2.056 (0.998)	1.429 (0.811)	0.037
	**Antihypertensive treatment (** ***n*** **)**		
	**Present (11)**	**Absent (28)**	
Carotid plaques (total count)	2.091 (2.119)	0.750 (1.266)	0.020
ABI	1.099 (0.136)	0.942 (0.145)	0.004
	**Hypolipidemics (** ***n*** **)**		
	**Present (2)**	**Absent (37)**	
Carotid plaques (total count)	4.000 (0)	0.973 (1.536)	0.009
Carotid plaques (max. thickness)	4.100 (1.556)	0.911 (1.568)	0.008
CVR-US	3.000 (0)	1.649 (0.919)	0.047
	**Diabetes (** ***n*** **)**		
	**Present (6)**	**Absent (33)**	
ABI	1.112 (0.132)	0.964 (0.153)	0.034
	**Prediabetes (** ***n*** **)**		
	**Present (11)**	**Absent (28)**	
Carotid plaques (total count)	2.000 (1.612)	0.786 (1.548)	0.036
Carotid plaques (max. thickness)	1.964 (1.935)	0.725 (1.499)	0.039
CVR-SCORE2	2.273 (0.786)	1.607 (0.786)	0.025
CVR-US	2.364 (0.942)	1.464 (0.838)	0.006
	**Insulin treatment (** ***n*** **)**		
	**Present (2)**	**Absent (37)**	
Carotid plaques (total count)	4.000 (0)	0.973 (1.536)	0.009
CVR-US	3.000 (0)	1.649 (0.919)	0.047
	**Anti-Jo-1 positivity (** ***n*** **)**		
	**Present (10)**	**Absent (29)**	
SCORE	0.700 (0.949)	4.034 (3.156)	0.002
	**Anti-Jo-1 positivity (** ***n*** **)**		
	**Present (10)**	**Absent (29)**	
SCORE2	2.500 (2.593)	14.345 (17.247)	0.039
mSCORE	1.050 (1.423)	6.052 (4.735)	0.002
CIMT	0.636 (0.120)	0.750 (0.147)	0.034
CVR-SCORE	1.000 (0)	1.448 (0.632)	0.032
CVR-SCORE2	1.100 (0.316)	2.034 (0.823)	0.001
CVR-mSCORE	1.000 (0)	1.724 (0.882)	0.014

*Data are presented as mean (standard deviation). ABI, ankle brachial index; CIMT, carotid intima-media thickness; CV, cardiovascular; CVR, cardiovascular risk; CVR-mSCORE, cardiovascular risk estimated according to the calculated mSCORE; CVR-SCORE, cardiovascular risk estimated according to the calculated SCORE; CVR-SCORE2, cardiovascular risk estimated according to the calculated SCORE2; CVR-US, cardiovascular risk based on carotid ultrasound examination (plaques, CIMT); Jo-1, anti-histidyl-tRNA synthetase; PWV, pulse wave velocity; SCORE, Systematic COronary Risk Evaluation; US, ultrasound (examination)*.

#### Traditional Risk Factors

Arterial hypertension was the main factor associated with a higher SCORE (as expected), carotid plaque count and thickness, PWV, and an overall CV risk (*p* < 0.05 for all). Antihypertensive treatment was associated with an increase in carotid plaque count (*p* = 0.020) but a more favorable (higher) ABI (*p* = 0.004). Treatment with lipid-lowering drugs was associated with an increase in carotid plaque count and thickness (*p* = 0.009, *p* = 0.008) and higher CVR-US (*p* = 0.047). Diabetes was associated with lower (worse) ABI values (*p* = 0.034), while prediabetes with a higher count (*p* = 0.036) and thickness (*p* = 0.011) of carotid plaques, and also with a worse (higher) CVR-US (*p* = 0.006). Patients treated with insulin had a higher count of carotid plaques (*p* = 0.009) and a higher CVR-US (*p* = 0.047). Peroral antidiabetic treatment and smoking had no influence on the CV risk or US pathological findings. However, some of these observations need to be interpreted with caution, since there were only two IIM patients treated with hypolipidemics or insulin and three smokers in our IIM cohort (Table 9).

#### Autoantibodies

ANA positivity was found in 24 (62%) of our IIM patients, while other most prevalent antibodies included anti-Jo-1 in 10 (26%) and anti-Ro-52 in 12 (31%) patients. Other autoantibodies were sporadically prevalent, and therefore were not analyzed (Table 1). Only anti-Jo-1 positivity was associated with a lower (better) CIMT (*p* = 0.034) and lower SCORE and its modifications, as well as the estimated CV risk based on these scoring systems: SCORE (*p* = 0.002), SCORE2 (*p* = 0.038), and mSCORE (*p* = 0.002) (Table 9).

On further examination, compared to anti-Jo-1 positive patients (*n* = 10), anti-Jo-1 negative patients (*n* = 29) showed a trend toward lower percentage of females (76% vs. 100%, *p* = 0.086), significantly higher age (*p* = 0.006), higher BMI (*p* = 0.013), higher prevalence of arterial hypertension (62% vs. 0%, *p* < 0.001) and a trend toward higher prevalence of diabetes (21% vs. 0%, *p* = 0.118). No other traditional CV risk factors significantly differed between these two groups. The presence of ILD was numerically higher in the anti-Jo-1 positive group as expected, but did not reach statistical significance (60% vs. 35%, *p* = 0.157). However, the anti-Jo-1 positive group had significantly higher levels of CK, myoglobin, and higher ESR compared to the anti-Jo-1 negative group (*p* < 0.05 for all). Additionally, disease activity (MITAX), tissue damage (MDI), muscle strength (MMT-8), treatment with MTX and AZA, as well as the cumulative dose and exposure time of GC were comparable in both groups (*p* > 0.05 for all, data not shown).

#### Clinical Manifestations

Due to potential bias by a very high or very low prevalence of some clinical manifestations, such as muscle weakness (88%), cardiac involvement (8%), skin rash and arthritis (13% each), only parameters with a prevalence of 20–80% were included in the analysis. ILD was present in 41%, RP in 26% and MH in 21% of IIM patients (Table 1). Nevertheless, no association between ILD, RP or MH, and the CV risk or findings of CV examination were detected in our IIM cohort.

#### Treatment

Similarly to clinical manifestations, analysis of a highly prevalent treatment [such as GC (92%)] or an infrequent treatment [such as cyclophosphamide (5%), leflunomide (5%), or mycophenolate mofetil (3%)] could cause significant bias (Table 1). Therefore, we only assessed the current (long-term) therapy with MTX and AZA; however, we have found no significant associations with the CV risk or the CV examination results. Because of the known potential negative impact of GC therapy on CV risk, we focused on the long-term GC therapy. We tested the association of the cumulative dose of GC (assessed as the prednisolone equivalent dose) and the time of exposure to GC. No significant association between CV risk and the GC cumulative dose was found. The only significant observation was the association of the exposure time to GC therapy with the total count of carotid plaques (p < 0.001) and with the maximum carotid plaque thickness (*p* = 0.003) on US.

#### Multivariate Analysis Models

Only variables with *p* < 0.25 from the univariate analysis were included in the subsequent multivariate analysis. Variables with *p* < 0.05 were considered significant. The age of the patients was the most significant factor, which affected most of the parameters (SCORE and its modifications, PVW, CIMT and the total count of carotid plaques). Exposure time to GC therapy was another significant factor that affected the total count and maximum thickness of the carotid plaques. Other significant predictors in the multivariate analysis included TC and AI (for ABI), mean arterial pressure (for PWV), disease duration (for the total count of carotid plaques), and chemokines (MCP-1 and MIP-1b for CIMT, and MIP-1a for PWV). Detailed data are shown in Table 10.

**Table 10:** Association of cardiovascular risk and subclinical atherosclerosis markers with disease-specific features, body composition parameters, and traditional risk factors in IIM, based on multivariate regression analysis.

**Correlated parameters**	**Disease features, CV risk factors, body composition**	**b**	***p*** **-value**
SCORE	Age	0.121	0.016
SCORE2	Age	0.568	0.023
mSCORE	Age	0.162	0.043
CIMT	Age	0.006	<0.001
	MCP-1	−0.001	0.013
	MIP-1b	0.0006	0.003
ABI	TC	0.055	0.016
	AI	−0.042	0.003
PWV	Age	0.059	0.036
	MIP-1a	0.667	0.046
	TC	0.607	0.030
Carotid plaques (total count)	Age	0.034	0.042
	Disease duration	−0.128	0.037
	GC-exposure time	0.0008	<0.001
Carotid plaques (maximum thickness)	GC-exposure time	0.0004	0.003

*ABI, ankle brachial index; AI, atherogenic index of plasma; b, regression beta coefficient; CIMT, carotid intima-media thickness; CV, cardiovascular; GC, glucocorticoids; MAP, mean arterial pressure; MIP-1a, macrophage inflammatory protein-1a; mSCORE, modified Systematic COronary Risk Evaluation; PWV, pulse wave velocity; SCORE, Systematic COronary Risk Evaluation; TC, total cholesterol*.

## Discussion

This is preliminary data from a cross-sectional study on CV risk in IIM compared to sex- and age-matched healthy controls. To our knowledge, it is the first study in IIM to include lipid profile, body composition (BIA and DXA), three non-invasive examinations of subclinical atherosclerosis, and assessment of CV risk (SCORE, SCORE2, and mSCORE).

To date, there are only limited data on CV morbidity and mortality in IIM. CV diseases are the leading cause of mortality in IIM (13), especially due to accelerated ATS of coronary arteries and myocarditis (15). CV diseases account for one-fifth of hospitalisations and double the risk of death in DM patients (46). Overall, IIM patients have more than double the CV risk as the general population (12). A recent study RI.CAR.D.A. (47) described significantly higher aortic stiffness and subclinical ATS in ASS patients compared to HC. Contrarily, we did not demonstrate any significant increase in the CV risk in our IIM patients, despite slightly worse findings on CV examination (carotid ultrasound, CIMT, ABI, and PWV). There was no significant difference between IIM and HC in the CV risk assessed by SCORE or CV examination, probably due to a relatively short disease duration (median 4.8 years), mild disease activity (median MITAX 0.13), and low disease damage (median MDI 0.05).

Due to the lack of recommendations or adequate tools for evaluating CV risk in rare rheumatic diseases, EULAR issued recommendations to multiply SCORE by the coefficient 1.5 for patients with inflammatory arthropathy (18). Similarly, multiplication of Framingham risk score by 2 is recommended for patients with SLE (48). Herein, we calculated CV risk using the recently validated SCORE2 (17) for the general population, but also included the original SCORE (16). For the IIM cohort, we used mSCORE (18) since the Framingham risk score is currently not widely used in Europe (49). Reclassification after incorporating carotid ultrasound examination showed that none of these scoring systems is accurate and was significantly superior or inferior in IIM. Numerically, the proportion of IIM patients reclassified to the higher CV risk level was the highest when originally estimated by SCORE (35.9%) and the lowest when originally estimated by SCORE2 (15.4%); however, this difference was not statistically significant. On the other hand, using SCORE2 in our HC resulted in significantly lower reclassification of CV risk level compared to SCORE algorithm (5.1% vs. 28.1%, respectively). Similarly, the RI.CAR.D.A. study demonstrated the limitations of SCORE and mSCORE in ASS patients for estimating CV risk, in comparison to cf-PWV and carotid ultrasound examination. Nevertheless, SCORE and mSCORE were not significantly different between ASS and the control group, which is consistent with our results (47). Recently, another similar study compared CV risk and clinical examination in patients with psoriatic arthritis, and recommend combining scoring tools with carotid ultrasound examination (CIMT) (50). To date, no studies have validated the CV risk estimation tools currently used in IIM, except for RI.CAR.D.A.

Increased arterial stiffness and CIMT have previously been described in PM and other rheumatic diseases compared to HC, while PM patients had milder arterial impairment (by PWV) compared to patients with systemic sclerosis (51). Another study demonstrated a tendency of increased CIMT and PWV in IIM compared to HC (52). Even adult patients with a history of juvenile DM may exhibit worse subclinical ATS (53).

When comparing the prevalence of traditional CV risk factors, there were no significant differences between our IIM cohort and HC. However, diabetes was non-significantly more prevalent in our IIM group. Studies on IIM reported significantly higher BMI and more prevalent arterial hypertension, diabetes, hypercholesterolemia (54–56), and metabolic syndrome compared to the general population (57).

While systemic inflammation in rheumatic diseases is generally related to atherogenesis (58), our data did not indicate more severe subclinical ATS in IIM, even after adjusting for traditional risk factors. A possible explanation is the small sample size and mild disease activity in our IIM patients. Generally, CRP is only slightly elevated in IIM patients (59, 60). Increased systemic inflammation is also associated with anti-Jo-1 positivity (60, 61). Anti-Jo-1 is the most common antibody associated with ASS (62, 63), and has also been implicated in the pro-inflammatory response (64). However, anti-Jo-1 is not specifically associated with an increased ATS-related CV risk. Surprisingly, in our IIM cohort, anti-Jo-1 positivity was associated with a lower SCORE and a decreased CV risk. Moreover, despite a higher prevalence of ILD and levels of muscle damage markers and ESR in the anti-Jo-1 positive compared to anti-Jo-1 negative patients, disease activity and tissue damage were similar in both groups. In contrast, anti-Jo-1 negative patients had more prevalent CV risk factors, such as arterial hypertension and diabetes, higher age and BMI, which may be the cause of the increased CV risk in our anti-Jo-1 negative patients.

Regarding clinical manifestations, ILD negatively influences physical condition, physical activity and thus probably worsen CV status (45). Additionally, previous studies showed that RP could be associated with a higher risk of CV disease (65). Nevertheless, no association between disease-specific clinical features and worse CV risk was demonstrated in our study.

Interestingly, several chemokines and cytokines were associated with an increased CV risk and markers of subclinical ATS on bivariate or multivariate analysis: MIP-1a, MIP-1b, IP-10, MCP-1, and IL-9. MIP-1a (CCL3) participates in the chemotaxis of inflammatory cells, granulocyte activation, atherogenesis, and CV disease development (66, 67). It is a potential biomarker of CV diseases or a prognostic factor of CV events (68, 69). The association of PWV with MIP-1a levels in our IIM cohort seems to confirm its relation with CV risk. MIP-1b (CCL4) participates in the adhesion of monocytes to the endothelium, and its serum levels can be a predictive factor for CV events (70, 71). MCP-1 (CCL2) attracts monocytes, memory T cells, and dendritic cells to the inflammation site, and is important for the formation of atherogenic plaques via its chemotactic activity on monocytes, which penetrate into the subendothelial space and become foam cells (72). IP-10 (CXCL10) is stimulated by interferon γ (IFN-γ) and attracts cells of monocyte/macrophage system and others. IP-10 levels and other chemokines are related to adverse remodeling of the myocardium accompanying ventricular dysfunction and heart failure (73, 74). IL-9, a cytokine with pleiotropic functions produced by multiple cell types, Th-9 specifically, is involved in various autoimmune and allergic inflammation (75). Elevated IL-9 levels were described in patients with atherosclerotic disease of the carotid and coronary arteries (76), and an increased Th-9 count could be involved in atherogenesis (75).

We also assessed the potential influence of immunosuppressive treatment on CV risk. The role of GC in CV risk in the rheumatic population is rather controversial. On the one hand, it suppresses inflammation, leading to endothelial dysfunction and damage; on the other hand, GC promotes the traditional risk factors (77). A long-term GC therapy at high doses (>7.5 mg of prednisolone daily) in patients with RA potentially increases CV risk. Nevertheless, the effect of low doses is not clear (78). Herein, we observed an association of the GC exposure time with carotid plaques. However, there was no association with the cumulative dose. This suggests that rapid dose tapering and discontinuation of GC are more important than the doses *per se*, especially during the manifestation and relapse of the disease. MTX and AZA were the most used csDMARDs in our cohort, but neither affected CV risk or subclinical ATS parameters. MTX has been even described as a potentially cardioprotective drug in rheumatic diseases (79), although this effect was not confirmed in the general population with an increased CV risk (80). The effect of AZA and other csDMARDs has not been sufficiently described. A Canadian study described a potential protective effect of non-steroid immunosuppressive drugs on the risk of arterial events in IIM patients (81). Nevertheless, therapy with GC-sparing agents (csDMARDs) should be preferred to GC monotherapy in IIM (82).

Finally, the main limitation of our study is a small IIM cohort, which precluded analysis on individual subsets. We used only non-invasive methods for CV examination, which could be inaccurate. Carotid plaque assessment, instead of CIMT, is preferred as a predictive marker for CV diseases or events (39), but CIMT monitoring could be beneficial in estimating CV risk (83). However, the cutoffs for the plaque thickness or count have not been clearly defined; therefore, threshold of high-risk plaques (plaque thickness >1.9 mm) and a risk plaque count >1 (total plaque thickness >1.9 mm) in our study were based on previous studies (44).

## Conclusion

CV risk in our cross-sectional cohort of IIM patients does not appear to be significantly increased compared to HC. Validated scoring systems for CV screening (SCORE2, mSCORE) comparably underestimate or overestimate CV risk in IIM patients with respect to subclinical atherosclerosis. Since there are currently no established tools for CV risk screening in IIM, both of these scoring systems, as well as clinical examination of subclinical atherosclerosis, should be considered for assessing CV risk in IIM patients. Regarding the potentially adverse effect of long-term therapy with glucocorticoids, and the potentially favorable effect of methotrexate on CV risk, glucocorticoid therapy should be as short as possible with preferable use of corticoid-sparing DMARDs.

## Data Availability Statement

The original contributions presented in the study are included in the article/Supplementary Material, further inquiries can be directed to the corresponding author/s.

## Ethics Statement

The studies involving human participants were reviewed and approved by Ethics Committee of the Institute of Rheumatology in Prague with reference numbers 10114/2016 and 9406/2017. The patients/participants provided their written informed consent to participate in this study.

## Author Contributions

SO, HS, LS, JV, MV, and MT designed the study. SO, HS, JKu, VT, OM, ZK, MSa, EC, JKv, MSp, BH, KP, LS, HM, MV, and MT collected patient data. HS performed the laboratory analysis. SO, MSp, and BH performed the body composition analysis. JKu, VT, OM, ZK, MSa, EC, and JKv performed the cardiovascular examination. PC and MR performed the statistical analysis. SO and MT prepared the original draft of the manuscript. All authors critically interpreted the results, reviewed the draft version, and approved the final manuscript.

## Funding

This work was supported by the Ministry of Health of the Czech Republic [023728, NV18-01-00161A]; Ministry of Education Youth and Sports of the Czech Republic [SVV 260523], Charles University Grant Agency [GAUK 312218], and Biobanks and Biomolecular Resources Research Infrastructure Consortium [BBMRI-CZ LM2018125]. There is no financial support or other benefits from commercial sources for the work reported in the manuscript.

## Conflict of Interest

The authors declare that the research was conducted in the absence of any commercial or financial relationships that could be construed as a potential conflict of interest.

## Publisher's Note

All claims expressed in this article are solely those of the authors and do not necessarily represent those of their affiliated organizations, or those of the publisher, the editors and the reviewers. Any product that may be evaluated in this article, or claim that may be made by its manufacturer, is not guaranteed or endorsed by the publisher.

## Glossary

A, arthritis; ABI, ankle brachial index; AI, atherogenic index of plasma; ANA, antinuclear antibodies; apo-A, apolipoprotein-A; AS, ankylosing spondylitis; ASS, antisynthetase syndrome; AST, aspartate aminotransferase; AZA, azathioprine; BCM, body cell mass; BDI-II, Beck's Depression Inventory-II; BF, body fat; BIA, bioelectrical impedance; BMC, bone mineral content; BMI, body-mass index; BMR-DXA, basal metabolic rate measured by dual energy X-ray absorptiometry; cf-PWV, carotid-femoral pulse wave velocity; CI, cardiac involvement; CIMT, carotid intima-media thickness; CK, creatine kinase; CPA, cyclophosphamide; CRP, C-reactive protein; CSA, cyclosporin A; CV, cardiovascular; CVR, cardiovascular risk; CVR-mSCORE, cardiovascular risk estimated according to the calculated mSCORE; CVR-SCORE, cardiovascular risk estimated according to the calculated SCORE; CVR-SCORE2, cardiovascular risk estimated according to the calculated SCORE2; CVR-US, cardiovascular risk estimated according to the carotid ultrasound examination (total plaque count, plaque thickness, carotid intima-media thickness); DM, dermatomyositis; DXA, dual-energy X-ray absorptiometry; ECM, extracellular mass; ECM/BCM, extracellular mass/body cell mass ratio; ECW, extra-cellular water; ESR, erythrocyte sedimentation rate; EULAR, European Alliance of Associations for Rheumatology; FFM, fat free mass; FIS, Fatigue Impact Scale; GC, glucocorticoids; HAP, Human Activity Profile; HAQ, Health Assessment Questionnaire; HC, healthy controls; HDL, high-density lipoprotein; IBM, inclusion body myositis; ICW, intra-cellular water; IIM, idiopathic inflammatory myopathies; IL, interleukin; ILD, interstitial lung disease; IMNM, immune-mediated necrotizing myopathy; IoRP, Institute of Rheumatology in Prague; IP-10, interferon-gamma-induced protein 10 (CXCL10); IQR, inter-quartile range; Jo-1, anti-histidyl-tRNA synthetase; Ku, anti-Ku (against the nuclear DNA-protein kinase subunit); LBM, lean body mass; LBM-DXA, lean body mass tissue measured by dual energy X-ray absorptiometry; LD, lactate dehydrogenase; LD, lactate dehydrogenase; LEF, leflunomide; MAF, Multidimensional Assessment of Fatigue Scale; MAP, mean arterial pressure; MCP-1, monocyte chemoattractant protein 1; MDA-5, anti-antigen associated with melanoma differentiation; MDI, myositis damage index; MMF, mycophenolate mofetil; MH, mechanic's hands; Mi-2, anti-nuclear helicase 218/240 kDa; MIP, macrophage inflammatory proteins; MIP-1a, macrophage inflammatory protein-1a; MITAX, Myositis Intention to Treat Activity Index; MMT-8, manual muscle testing of 8 muscle groups; mSCORE – modified Systematic COronary Risk Evaluation; MTX, methotrexate; MW, muscle weakness; NXP-2, anti-nuclear matrix protein; OD, esophageal motility disorder; PDGF, platelet-derived growth factor; PM, polymyositis; PM-Scl, anti-Pm-Scl (anti-core complex 11-16 proteins); PRO, Patient Reported Outcome; PsA, psoriatic arthritis; PWV, pulse wave velocity; RA, rheumatoid arthritis; RANTES, regulated on activation/normal T cell expressed and secreted; RMR, resting retabolic rate; RNP, anti-ribonucleoprotein; Ro, anti-Ro (52/60kDa, against cytoplasmic RNA and associated peptides); RP, Raynaud's phenomenon; RSMI, relative skeletal muscle index; SAE, anti-SUMO1 (small ubiquitin-like activating enzyme); SCORE – Systematic COronary Risk Evaluation; SD, standard deviation; csDMARDs, conventional synthetic disease modifying antirheumatic drugs; SF-36, Medical Outcomes Study 36-item Short Form Health Survey; SLE, systemic lupus erythematosus; SR, skin rash; SRP, anti-signal recognition particles; TBW, total body water; TC, total cholesterol; TIF-1, anti-transcription factor-1; TNF, tumor necrosis factor; US, ultrasound (examination).
