# Supplementary material for: Cardiovascular Risk in Myositis Patients Compared to the General Population: Preliminary Data From a Single-Center Cross-Sectional Study
Source: Front Med (Lausanne). 2022 May 3;9:861419. doi: 10.3389/fmed.2022.861419 (PMC9118331; doi:10.3389/fmed.2022.861419)
Supplement: Supplementary file 1 [file Data_Sheet_1.docx]

**
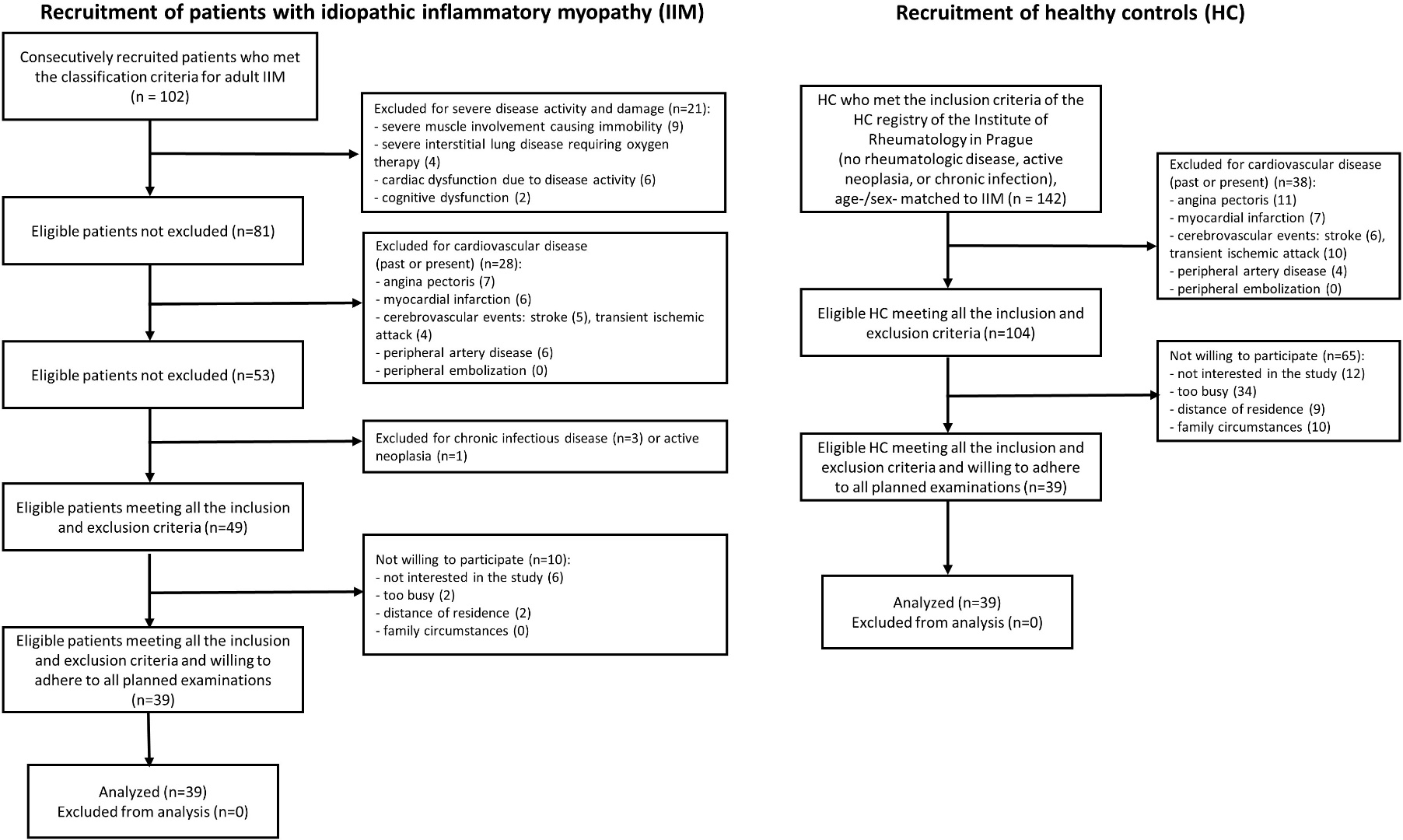
SUPPLEMENTARY FIGURE 1 | Recruitment of patients with idiopathic inflammatory myopathy (IIM) and healthy controls (HC)**

**SUPPLEMENTARY FIGURE 2 |** Reclassification of the cardiovascular risk category based on the cardiovascular risk scoring systems (i.e., < 5%, 5-10%, and > 10% risk of fatal events) to the cardiovascular risk category according to ultrasound (US) markers of subclinical atherosclerosis (carotid intima-media thickness, and carotid plaques) (i.e., low, intermediate, and high risk) in patients with idiopathic inflammatory myopathies (IIM): A (SCORE vs. US), B (SCORE2 vs. US), C (mSCORE vs. US) and healthy controls (HC): D (SCORE vs. US), E (SCORE2 vs. US). *low, <2.5% (for <50 years of age), <5% (for 50-69 years of age), <7.5% (for ≥70 years of age); *intermediate, 2.5-7.5% (for <50 years of age), 5-10% (for 50-69 years of age), 7.5-15% (for ≥70 years of age); *high, ≥7.5% (for <50 years of age), ≥10% (for 50-69 years of age), ≥15% (for ≥70 years of age)
